# Sequencing complete plasmids on Oxford Nanopore Technologies sequencers using *R2C2* and *Chopper*

**DOI:** 10.1371/journal.pone.0345168

**Published:** 2026-04-10

**Authors:** Kayla D. Schimke, Christopher Vollmers

**Affiliations:** Department of Biomolecular Engineering, University of California Santa Cruz, Santa Cruz, California, United States of America; Indian Institute of Technology Kanpur, INDIA

## Abstract

Plasmids are ubiquitous tools in molecular biology which are used for a large variety of experiments within academic and commercial labs. Both new and old plasmids have to undergo sequencing-based analysis to determine whether or not they are functional, i.e., contain the correct insert in the correct backbone. While traditional Sanger sequencing based analysis was most often limited to the inserts, new high-throughput sequencing based methods and services can now provide the complete sequence of a plasmid. Currently available methods and services vary in throughput and cost. Here, we adapted the Oxford Nanopore Technologies-based R2C2 sequencing method to – rapidly and at low cost – sequence complete plasmids, either individually or in a pool. We also developed an analysis pipeline, Chopper, that produces full-length plasmid sequences. We tested our workflow with commonly used plasmids we ordered from Addgene and produced highly accurate sequences for each plasmid from both their individual and pooled sequencing runs.

## Introduction

Plasmids are fundamental tools in molecular biology serving as vectors for recombinant DNA. Following their assembly, as well as periodically after, their DNA sequence has to be validated to ensure they are functional and retain their functionality over time.

Traditionally, plasmid sequencing has been performed using Sanger sequencing and has focused on the specific inserts in the plasmids, like the coding sequence of a protein, leaving the often reused backbones of the plasmids – promoters, origins of replication, antibiotic resistances genes – unobserved and subject to potential mutations that might affect their functions.

High throughput sequencing can address this by sequencing and assembling entire plasmids. Multiple institutions, including Applied Biological Materials Inc, seqWell, Massachusetts General Hospital Center for Computational and Integrative Biology, and CD Genomics, offer plasmid sequencing on Illumina platforms. While short read sequencing is accurate, the turnaround time is less than ideal for quick validations (>2 weeks) and the reliance on fragmenting before sequencing could prevent complete assembly – especially if the plasmid contains repetitive sequences much longer than Illumina read length. Indeed, some of the previously mentioned institutions charge a fee for assembling and annotating a plasmid sample and still cannot guarantee complete assemblies.

To address this shortcoming, several sequencing providers have turned to long read sequencing to ensure they capture full length plasmids; CD Genomics offers sequencing with Pacific Biosciences (PacBio) SMRT technology specifically for large plasmids, while Plasmidsaurus sequences plasmids exclusively with Oxford Nanopore Technologies (ONT). These facilities offer a quicker turnaround time and, in the case of Plasmidsaurus, a very low price (Table 1).

**Table 1:** Commercial plasmid sequencing statistics. *additional cost for assembly and annotation **dependent on plasmid size. *** per well in 96 well plate. (Data collected Jan 2024).

Company	Technology	Input requirements	Price per sample	Turnaround time
ABM	Illumina	1 - 50 ng	$315*	4-6 weeks
seqWell	Illumina	600 - 1500 ng ***	NA	2 weeks
MGH CCIB	Illumina	1400 - 2275 ng	$56.16 - $70.20	5 - 8 business days
CD Genomics	Illumina/PacBio	1400 - 2275 ng	$270**	15 business days
Plasmidsaurus	ONT	300 - 2000 ng **	$15 - $60**	1 business day

Despite opportunities to outsource plasmid sequencing, there are several reasons why sequencing plasmids in house might be preferable to academic and commercial labs. These include using plasmid sequencing for training purposes, intellectual property concerns when outsourcing, sequencing plasmids that contain libraries of inserts, or simply the lack of availability of service providers.

ONT sequencing, using a MinION or P2 Solo, makes it possible for most labs to sequence complete plasmids in-house. There are also web- or self-hosted computational tools like PlasCAT [1] or ONT-developed wf-clone-validation (https://github.com/epi2me-labs/wf-clone-validation) for the assembly of plasmid sequences from ONT data. Furthermore, there already exist protocols for the preparation and sequencing of plasmids on ONT sequencers that perform very well. Initial ONT-based methods sequenced targeted complex synthetic constructs that were just part of entire plasmids [2]. These were followed by other ONT-based methods that achieved error-free sequencing of entire individual plasmids [3] and tagmentation based, multiplexed workflows like Circuit-seq [4] and onRamp [5] which sequenced large pools of entire plasmids. While approaches sequencing individual plasmids [3] achieved error-free plasmid sequences, using an entire flow cell per plasmid puts the cost of sequencing one plasmid at hundreds of dollars. Multiplexed workflows [4,5] dramatically reduced this cost to a few dollars per plasmid. However, this reduced per plasmid cost is only realized when many plasmids are sequenced at once which is not necessarily the case for many molecular biology laboratories.

Here, we set out to develop a method that could economically prepare and sequence individual or small pools of plasmids. We also wanted to develop an analysis tool to create error-free plasmid sequences from the resulting data.

To this end, we modified our R2C2 protocol [6] to use phi29-polymerase based rolling circle amplification (RCA) and T7 Endonuclease based debranching of the RCA product to generate linear dsDNA suitable for ONT sequencing. To reduce sequencing cost, we sequenced this dsDNA in our lab on an ONT P2 Solo using previously used flow cells and a modified library preparation protocol. We then processed the resulting data with a modified version of the established C3POa tool to parse individual sequencing reads and the newly developed Chopper to generate highly accurate polished full-length plasmid sequences.

## Results

### Sequencing plasmids with a modified R2C2 workflow

We purchased four plasmids with known reference sequences from Addgene:

pCSCMV:tdTomato (tdTomato), lentiCRISPR v2 (lentiCRISPR), pSpCas9(BB)-2A-Puro (PX459) V2.0 (pSpCas9), pcDNA3.1-GFP(1–10) (pcDNA3.1). We chose these plasmids because they covered a range of lengths from 5kb up to 15kb, the long end of which might push the limits of the R2C2 protocol. Further, tdTomato contained a tandem repeat and pSpCas9 contained two long homopolymers which might present challenges for consensus generation by C3POa and polishing by Chopper. Finally, these plasmids represent some of the most commonly ordered plasmids from Addgene which means they are representative of plasmids actively used in molecular biology labs.

**Fig 1:**
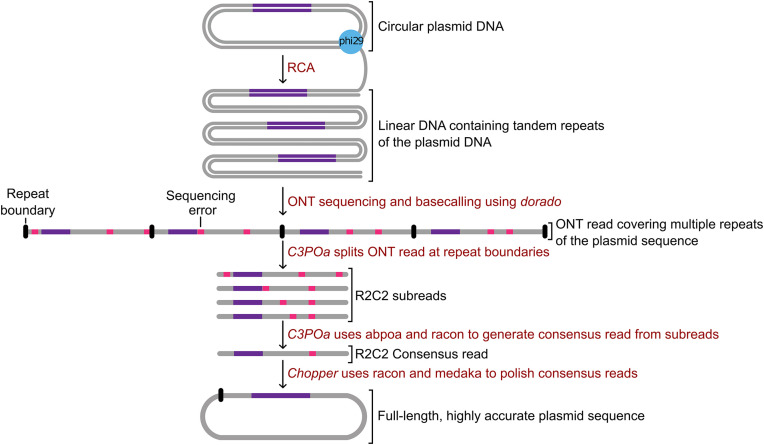
Plasmid processing, sequencing and analysis. Plasmids were processed with rolling circle amplification (RCA) to produce linear DNA containing multiple tandem repeats of the original plasmids. The linear DNA was prepped and sequenced on an ONT P2Solo. The resulting ONT data were then processed into ONT reads by dorado and consensus reads by an updated version of C3POa. Consensus reads and subreads originating from multiple reads covering the same plasmid were then combined into one highly accurate plasmid sequence using the newly developed Chopper.

We then used a modified version of R2C2 to prepare each plasmid for sequencing – both individually and as part of a pool. Because the plasmids were already circular, we omitted the Gibson assembly step of R2C2 and instead directly amplified the plasmids with Rolling Circle Amplification (RCA) followed by debranching of the RCA product with T7 Endonuclease I. In this way, we produced long dsDNA containing multiple copies of the plasmids as tandem repeats (Fig 1).

After RCA and debranching, we performed a gel size selection of the resulting dsDNA, keeping only dsDNA that was over 10 kb in length. The size-selected dsDNA for each individual plasmid and the pool was then prepared for sequencing on an ONT P2 Solo using LSK114 ligation kits from ONT. Because this ONT library preparation represents the largest individual contributor to the cost of this plasmid sequencing workflow, we modified the protocol to use only 1/10th of the listed amount of sequencing adapter – the limiting reagent in the LSK114 kit.

For the actual sequencing runs we purposely sequenced on (in one case repeatedly) reused PromethION flow cells that had been used for previous experiments and were at the end of their useful life. Because the accuracy of consensus sequences based on ONT reads plateaus after a few hundred raw reads [7], we reasoned that we would only need a few hundred reads per plasmid.

### Generating and evaluating R2C2 consensus reads

After sequencing each individual plasmid and the pool, we basecalled the reads with dorado (v0.7.3, model dna_r10.4.1_e8.2_400bps_sup@v5.0.0) and obtained 58,209 ONT reads for tdTomato, 22,626 ONT reads for pcDNA3.1, 136,012 ONT reads for pSpCas9, 232,970 ONT reads for lentiCRISPR, and 193,117 ONT reads for the pool. The discrepancy in read numbers is related to the state of the flow cell before the experiment. Further, even though most of the DNA produced by R2C2 is 20–50kb long (S1 Fig) and we used gel-based size-selection on this DNA to select for molecules >= 10kb, the majority of ONT reads was shorter than 10kb. This is in line with previous R2C2 based studies that suggest that ONT sequencers have a strong preference for the short end of DNA molecules loaded for sequencing. This posed a challenge for downstream analysis.

**Fig 2:**
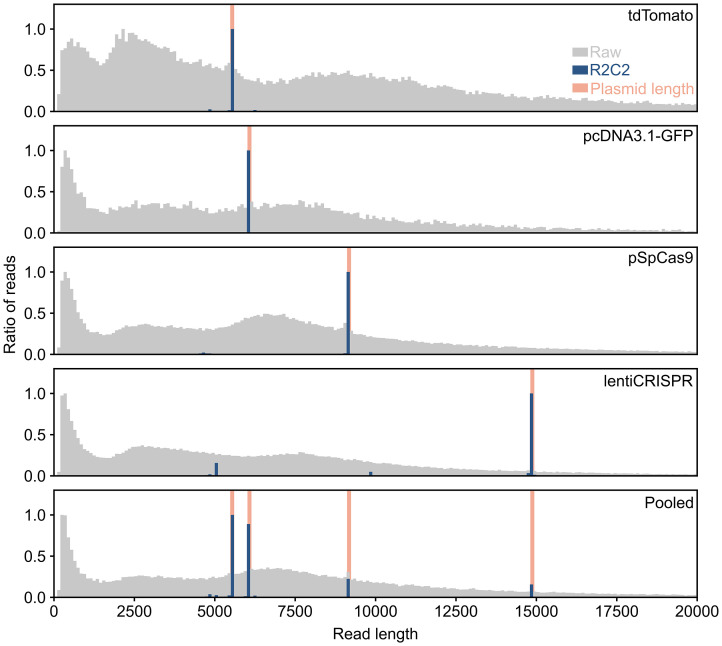
Sequencing plasmids with R2C2/C3POa. For each sequencing run, the read length distributions of the raw ONT sequencing data (grey) and R2C2 consensus reads (blue) are shown as histograms. The length of sequenced plasmids (orange) included in each run is also shown.

As the R2C2 method uses rolling circle amplification, these ONT reads contain tandem repeats of an original circular DNA molecule, we initially wanted to use Tidehunter which can recognize tandem repeats in ONT reads and, importantly, does not require any prior knowledge of the sequence of these tandem repeats. However, Tidehunter [8] requires at least two full tandem repeats to be present in the ONT read to detect it. This was a problem because the lentiCRISPR plasmid we aimed to sequence was about 15 kb long but only a small fraction of ONT reads were longer than 20kb (Fig 2) – shorter than the 30 kb it would take to cover two full repeats of this plasmid.

We therefore chose to adapt C3POa, the standard R2C2 analysis pipeline. The C3POa tool processes ONT reads covering DNA produced by the R2C2 method. It was originally designed to detect tandem repeats based on known splint sequences used to circularize molecules as part of the full R2C2 workflow. Since plasmids did not go through the full R2C2 workflow and therefore did not contain known splint sequences, we implemented a new option for C3POa: --nosplint or -ns, for raw data that does not contain a splint or other known sequence. When --nosplint is set, instead of a user-defined known splint sequence, C3POa uses a 200 bp anchor sequence taken from a few hundred bp into the ONT read itself to identify repeat boundaries.

Once C3POa has identified repeat boundaries, it splits the ONT read sequence at these boundaries to create R2C2 subreads (in the FASTQ format). If the R2C2 subreads are highly variable in length, potentially indicating splitting was faulty due to an internal repeat – present for example in the tdTomato plasmid – an ONT read is not processed further. If R2C2 subreads are of roughly equal length, C3POa creates a multiple sequence alignment of these R2C2 subreads using abpoa [9] to create a rough consensus which it then polishes using racon [10]. These polished R2C2 consensus reads (in FASTA format) are the final output of R2C2/C3POa.

We used this adapted C3POa version with the --nosplint option on the ONT reads of the different runs. This produced 37,617 R2C2 consensus reads for tdTomato, 10,361 R2C2 consensus reads for pcDNA3.1, 38,430 R2C2 consensus reads for pSpCas9, 23,541 R2C2 consensus reads for lentiCRISPR, and 65,971 R2C2 consensus reads for the pool. In all cases the length of the majority of these R2C2 consensus reads matched the length of the plasmid reference sequences (Fig 2).

A detailed analysis showed that the length 92% of tdTomato, 95% of pcDNA3.1, 88% of pSpCas9, 70% of lentiCRISPR R2C2 consensus reads fell within 1% of the length of their respective plasmid reference sequence. The relatively lower percentage of lentiCRISPR R2C2 consensus reads within 1% of the length the lentiCRISPR plasmid is likely due to the 15kb length of the plasmid approaching the length limitation of the R2C2 method. This showed that R2C2/C3POa can be used to generate full-length reads of plasmids from 5 to 15kb.

Once we had generated R2C2 consensus reads from all datasets using the updated C3POa, we evaluated their accuracy by aligning its output to the Addgene reference using minimap2. Accuracy was calculated by dividing the number of matches by the sum of matches, mismatches, and indels within the read alignment. The accuracy of the R2C2 consensus reads reached 99.86% ~(Q28) for tdTomato but declined with plasmid length approaching the raw ONT accuracy – shown by R2C2 Subread alignments for lentiCRISPR (Table 2).

**Table 2:** R2C2 consensus read characteristics for different plasmids.

Plasmid	Read type	Plasmid length (bp)	Median accuracy (%)	Average accuracy (%)
tdTomato	R2C2 Consensus	5,542	99.86	99.60
pcDNA3.1	R2C2 Consensus	6,077	99.73	99.5
pSpCas9	R2C2 Consensus	9.174	99.61	99.08
lentiCRISPR	R2C2 Consensus	14,874	99.54	98.48
lentiCRISPR	R2C2 Subreads	14,874	99.3	98.25

**Fig 3:**
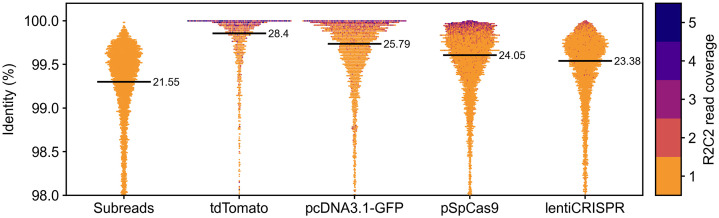
Evaluation of C3POa consensus accuracy. Accuracies of R2C2 subreads or R2C2 Consensus reads are shown for the indicated plasmids as swarmplots. R2C2 Subreads were from the lentiCRISPR plasmid. Median read accuracy is shown as black bars with the exact Q score listed to its right.

We assumed that the decrease in R2C2 consensus read accuracy could be attributed to subread coverage decreasing as plasmid length increases. To validate this assumption, we visualized read accuracy for each plasmid as swarmplots (Fig 3). As expected, shorter plasmids showed more highly accurate high-coverage reads than the longer plasmids.

Importantly, once only R2C2 consensus reads with subread coverage of 2 or more were considered, median read accuracy was around 99.9% for all four plasmids – regardless of plasmid lengths (tdTomato 99.96%; pcDNA3.1 99.97; pSpCas9 99.90%; lentiCRISPR 99.90%)

In summary, using a modified C3POa tool, we processed R2C2 plasmid sequencing data to generate accurate full-length plasmid sequences.

### Generating highly accurate plasmid sequences with Chopper

After R2C2 consensus calling, we wanted to generate error-free sequences for each plasmid from both individual and pooled runs. We also wanted to take advantage of the medaka tool developed by ONT which is updated alongside the ONT *dorado* basecaller models and achieves very high accuracy.

**Fig 4:**
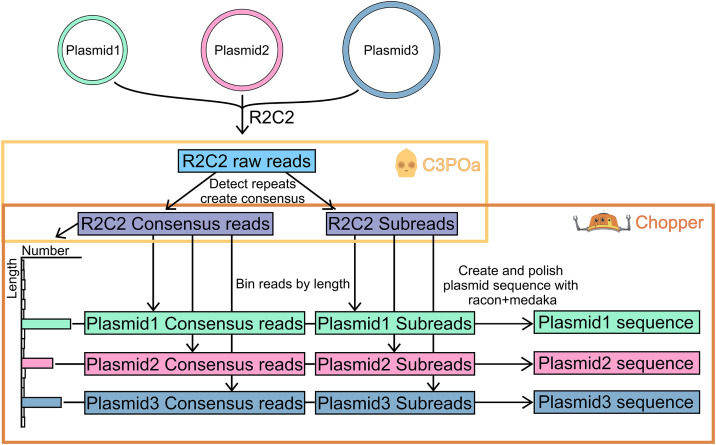
Chopper generates highly accurate plasmid sequences. Chopper accepts R2C2 consensus reads and their subreads produced by C3POa as input. It then bins R2C2 consensus reads by length to identify different plasmids in the sequencing data. For each length bin/plasmid, Chopper then generates a highly accurate plasmid sequence using racon and medaka for polishing.

To do so, we developed the Chopper tool (https://github.com/kschimke/Chopper/) (Fig 4).

Chopper processes R2C2 consensus and R2C2 subreads generated from ONT runs containing one or more plasmids in the following way:

To demultiplex R2C2 consensus reads originating from the sample plasmid, it separates these reads by length. To do so, Chopper bins R2C2 consensus reads by length and determines peak bins by comparing the size of the bins and finding local maxima. Chopper then separately processes the R2C2 consensus reads within each peak bin.

For each peak bin, Chopper identifies the R2C2 consensus reads which are most likely to be the most accurate. It does so by sorting the R2C2 consensus reads by 1) how many R2C2 subreads were used to create the R2C2 consensus read and 2) the average read quality – Q score calculated as -log_10_(estimated error rate)*10 and reported by the dorado basecaller for each base – of these R2C2 subreads.

Chopper then proceeds to polish the R2C2 consensus reads with the highest number or R2C2 subreads and highest Q scores. The exact number of R2C2 consensus reads to be polished can be set with the --iteration argument. To polish each R2C2 consensus read, Chopper first pads the read by appending the first half the read’s sequencing to the read’s end. This padding minimizes alignment errors due to the circular nature of the original sequenced molecules

Next, Chopper identifies the R2C2 subreads within the peak bin which are the most likely to be the most accurate. It does so by sorting the R2C2 subreads by Q scores. Chopper uses racon and medaka to polish each padded R2C2 consensus read. First, the R2C2 subreads (500 by default) with the highest Q scores are aligned to each padded R2C2 consensus read using minimap2. Chopper then uses racon [10] to polish the R2C2 consensus read based on those alignments. Second, the R2C2 subreads with the highest Q scores are aligned to each racon-polished padded R2C2 consensus read using minimap2. Chopper then uses medaka to polish the racon-polished R2C2 consensus read based on those alignments. Finally, Chopper trims the padded sequence off of the racon- and medaka-polished R2C2 consensus read to generate a full-length plasmid sequence.

We applied Chopper to the R2C2 consensus reads and subreads produced by C3POa for the sequencing runs of individual plasmids. Next, we wanted to determine if the output of *Chopper* is dependent on the R2C2 consensus read chosen to be polished. To do so, we ran *Chopper* on 100 R2C2 consensus reads for each peak bin using the --iteration argument. Further, to determine if the output of *Chopper* is dependent on the number of R2C2 subreads used for polishing these R2C2 consensus reads, we repeated this process using from 10–500 R2C2 subreads. We evaluated the accuracy of the *Chopper* output sequences for each condition and plasmid by aligning them to their respective plasmid references (as provided by Addgene) with minimap2 [11] using the same algorithm to calculate accuracy as we did for the R2C2 consensus reads.

**Fig 5:**
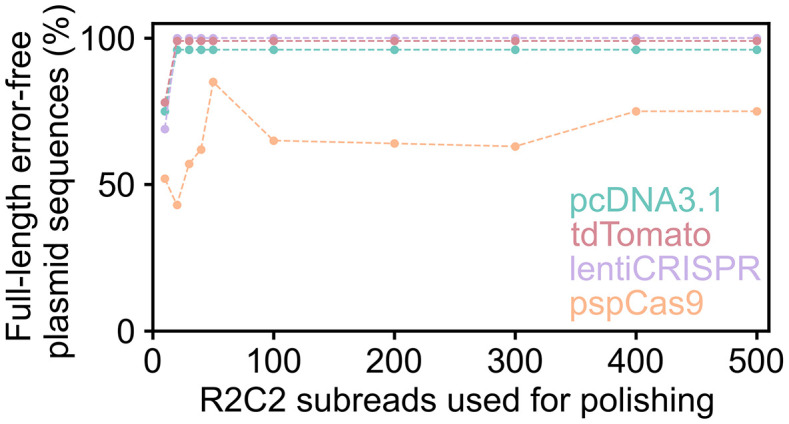
Chopper output accuracy depends on R2C2 subread coverage. Chopper was run on data from the 4 sequencing runs on individual plasmids. 100 R2C2 consensus reads were polished for each plasmid sequencing run (using the --iterations argument). Each R2C2 consensus read was polished with increasing numbers of R2C2 subreads (using the --subreads argument). With the exception of pSpCas9, Chopper produced almost entirely error-free sequences once more than 20 R2C2 subreads were used for polishing.

This analysis showed that Chopper produced almost exclusively error-free full-length plasmid sequences for the 100 R2C2 consensus reads for tdTomato, pcDNA3.1, lentiCRISPR R2C2 once at least 20 R2C2 subreads were used for polishing. However, Chopper produced at most 84 error-free full-length plasmid sequences for the 100 R2C2 consensus reads for the pSpCas9 plasmids, and it did so when 50 R2C2 subreads were used for polishing (Fig 5).

To determine if the performance of Chopper was the same when plasmids were run in a pool, we ran Chopper on the pooled sequencing run. Based on the previous experiment, we used 50 R2C2 subreads for polishing. Similarly to individual plasmid runs, Chopper produced error-free sequences for tdTomato, pcDNA3.1, and lentiCRISPR but struggled with pSpCas9–78/100 Chopper sequences for the pooled run were error-free and full-length (Table 3).

**Table 3:** Chopper generates highly accurate plasmid sequences. For each plasmid and run, the number of full-length error-free plasmid sequences out of the 100 sequences produced by Chopper (using 50 R2C2 subreads for polishing) is shown.

Sequencing Run	tdTomato	pcDNA3.1	pSpCas9	lentiCRISPR
Individual	99/100	98/100	86/100	100/100
Pool	100/100	99/100	78/100	100/100

**Fig 6:**
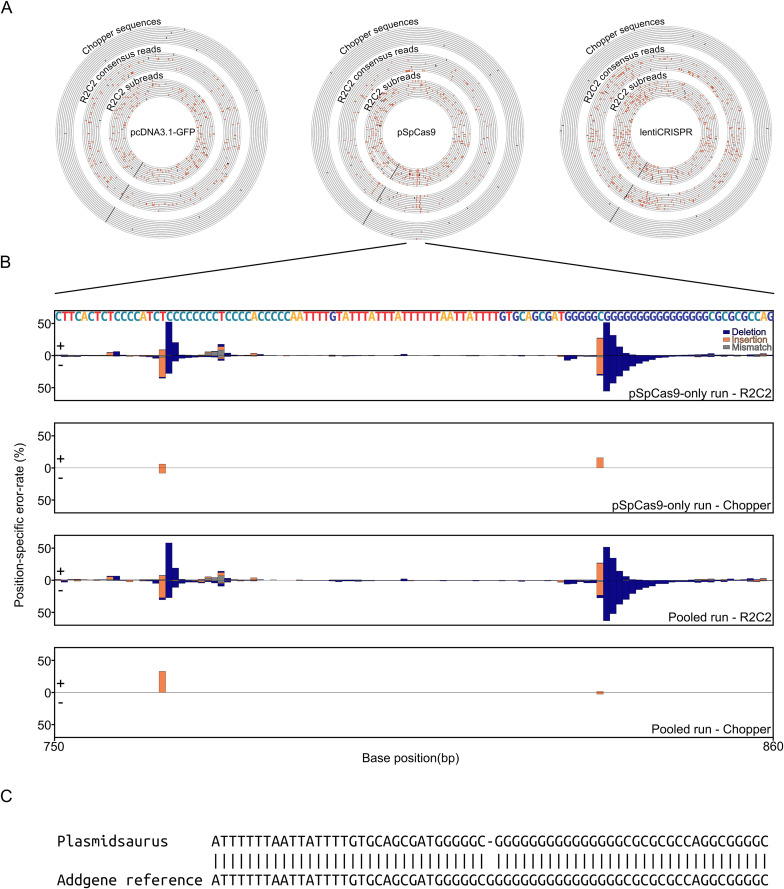
Chopper generates highly accurate plasmid sequences. R2C2 subreads, R2C2 consensus reads, and Chopper sequences are aligned to the indicated plasmid references. Mismatches and Indels and are indicated in red, alignment ends in black **B)**. The error-rate of R2C2 consensus reads and Chopper sequences at specific positions of the pSpCas9 plasmid. These error-rates are shown as stacked bargraphs for a region of the plasmid that contains two homopolymers which represent a source for systematic sequencing error. **C)** Pairwise alignment of the pSpCas9 plasmid reference and the sequence Plasmidsaurus produced for that plasmid.

To investigate what caused Chopper to struggle with the pspCas9 plasmid, we determined where R2C2 subreads, R2C2 consensus reads, and Chopper sequences contained errors. By visualizing the alignments of reads from the pcDNA3.1 and lentiCRISPR runs to their respective plasmids, we found that errors were mostly random in R2C2 subreads, reduced in R2C2 consensus reads, and entirely absent in Chopper sequences (Fig 6A, left and right).

For pSpCas9 however, R2C2 subreads, R2C2 consensus reads, and Chopper sequences all contained errors in two adjacent regions (Fig 6A, center).

These regions contained two homopolymers of eight Cs and sixteen Gs, respectively (Fig 6B). We quantified errors in R2C2 consensus reads across those two homopolymers and found a high level of systematic errors at those positions. Interestingly, Chopper, and by extension racon and medaka, was able to remove these systematic errors for most but not all polished sequences.

To determine if commercial plasmid sequencing services would struggle with these homopolymers as well, we sent all four plasmids in this study to Plasmidsaurus for sequencing and analysis. The resulting Plasmidsaurus assemblies of the tdTomato, pcDNA3.1, and lentiCRISPR plasmids were error-free, however the Plasmidsaurus assembly of the pSpCas9 plasmid contained a one base deletion in the longer G homopolymer (Fig 6C).

This indicated that even a dedicated commercial sequencing provider with a likely bespoke assembly pipeline struggles to correctly assemble long homopolymer stretches, thereby highlighting the remaining limitations caused by highly systematic errors produced by ONT sequencing technologies.

## Discussion

While it is difficult to argue with low cost and fast turnaround time for commercial whole plasmid sequencing, different labs might have different reasons to perform plasmid sequencing in-house. Heavily multiplexed plasmid sequencing workflows like onRamp [5] or Circuit-seq [4] make this possible but they only achieve a low cost per plasmid when many plasmids are sequenced at once.

To make it possible to prepare and sequence individual or small pools of plasmids for sequencing on ONT flow cells at low cost, we developed a modified R2C2 protocol. We processed plasmids 5–15kb in length with this method and sequenced them on previously used flow cells for a limited time. In this way, we generated tens to hundreds of thousands of R2C2 reads covering full-length plasmids with accuracies up to 99.86%. Investigating these individual R2C2 reads might be of use to labs screening plasmid pools where each plasmid molecule might contain a (slightly) different sequence. An example of this might be plasmids containing a library of CRISPR gRNA sequences.

However, most investigators will likely be interested in a single error-free full-length plasmid sequence. To provide these single sequences, we developed Chopper, which in this study produced plasmid sequences that – with the exception of very long homopolymers – were practically error-free.

In addition to accuracy, cost will be a consideration for investigators. We reduced the cost of the protocol by decreasing the volume of key ONT library preparation reagents used for each sample and heavily reduced the cost of flow cells by sequencing on previously used flow cells. If we estimate the cost of a used flow cell to be the reads it generated for plasmid sequencing divided by the total reads it generated in its life time multiplied by its initial cost, we arrived at about $7 [(100,000 plasmid reads/10,000,000 total reads)*$700 flow cell cost]. Creating around 100,000 reads for each plasmid is also likely to be excessive considering we only used 50 R2C2 subreads to create mostly error-free plasmid sequences. A few hundreds reads per plasmid would therefore probably have been sufficient.

Overall then, we estimate the cost of sequencing a single plasmid or a pool of plasmids in this way to be $70 – $10 for RCA, $12 for T7 treatment, $1 clean-up, $40 ONT prep (using diluted adapter) and $7 for the used flow cell. When sequencing a single plasmid, this is significantly more expensive than highly multiplexed academic methods or commercial service like Plasmidsaurus. However, it becomes cost competitive with Plasmidsaurus at pools of 4 or more plasmids. Importantly – due to the ability of the R2C2/C3POa/Chopper workflow to demultiplex plasmids based on their R2C2 consensus read lengths – plasmids can be pooled before RCA, independent of their sequence content and without any barcoding or indexing requirement, as long as the lengths of the pooled plasmids are at least 1% different from each other. For plasmids 5–10kb in length that means plasmids that are at least 50–100nt apart in length could be pooled without concerns for their reads being conflated by Chopper.

However, there are some limitations to our approach. First, due to the nature of the R2C2 method, plasmids much longer than 10kb will be sequenced less efficiently than shorter plasmids. If sequenced in a pool with shorter plasmids, longer plasmids could also generate partial R2C2 consensus reads which in turn could interfere with accurate demultiplexing by Chopper. Another limitation is while the R2C2 method has been simplified and optimized by our lab over the last 7 years, it is still a multi-step protocol which involves an overnight incubation step. To sequence the resulting DNA, the method requires access to an ONT sequencer and to do so economically also requires used flow cells. Finally, the ONT dorado basecaller, C3POa, and Chopper computational tools require a computer equipped with a powerful GPU that is running a Linux operating system.

Consequently, data generation and analysis using our approach takes 2–3 days from sample to polished, complete plasmid sequence which is longer than tagmentation based Circuit-seq and OnRamp methods (~1 day). Further, our approach requires familiarity with advanced DNA preparation and sequencing techniques as well as with computational tools run using a command line interface.

Because of that, the method we present here is likely best suited for labs that need to sequence a small number of plasmids in house but perform other types of ONT based sequencing routinely.

## Methods

### Library preparation

We purchased four plasmids with known references from *Addgene*: pCSCMV:tdTomato, lentiCRISPR v2, pSpCas9(BB)-2A-Puro (PAX459) V2.0, pcDNA3.1-GFP(1–10). We pooled 125 ng of each plasmid to create a pool before R2C2 conversion. R2C2 was performed as in [7]

with the exception of skipping the circularization step. In short, the individual plasmids and plasmid pool were amplified with rolling circle amplification (RCA) using Phi29 polymerase (NEB) and random hexamer primers (Thermo).

We performed 5 reactions for each plasmid and the pool using 100 ng of input each: [5 uL Phi29 Buffer (10X,NEB, M0269S), 1 uL Phi29 Polymerase(NEB, M0269S), 2.5 uL dNTP (10 mM;NEB N0447S), 2.5 uL Random hexamer primers (10 uM, Thermo, SO181), 10 uL plasmid (10ng/ul), 29 uL ultra-pure water]. RCA was incubated at 30℃ for 16h. The RCA product was debranched by adding 2ul of T7 endonuclease I (NEB, M0302S) and incubating at 37℃ for 2h. The resulting debranched DNA was then pooled and cleaned using a Monarch PCR and DNA Cleanup Kit (NEB). The purified RCA product was size-selected on an agarose gel: DNA at 10 kb and above was excised from the gel. R2C2 DNA was extracted from gel fragments using a Monarch DNA Gel Extraction Kit (NEB).

ONT libraries were prepared from R2C2 DNA using the ONT ligation sequencing kit V14 (ONT SQK-LSK114) following the manufacturer’s protocol while reducing the amount of Ligation Adapter to 1/10th of the recommended amount (0.5ul). The libraries were then sequenced on an ONT PromethION flow cell (R10.4.1) on a P2Solo for about 1 hour. Each flow cell was previously used for other experiments and reloaded with the plasmid R2C2 DNA after washing.

### Analysis

Raw nanopore sequencing data in the POD5 file format were basecalled using the *dorado* (v0.7.3) basecaller with the “sup” setting which uses the most accurate basecalling model (dna_r10.4.1_e8.2_400bps_sup@v5.0.0) to generate FASTQ files. FASTQ were then processed with C3POa (v3.2) to generate R2C2 consensus reads (FASTA format) and R2C2 subreads (FASTQ format). C3POa was run with default settings with the exception of the newly added -ns flag which indicates that the raw reads do not contain a known splint sequence.

C3POa splits raw ONT reads into R2C2 subreads and then combines those subreads into more accurate R2C2 consensus reads. Both R2C2 consensus and R2C2 subreads produced by individual and pooled plasmid runs were then processed by Chopper to generate 100 polished plasmid sequences for each size bin (-i 100). Cutoff percent for peaks (-t) was set to 10 for individual and and 2 pooled runs. For Fig 5, Chopper was run repeatedly with different numbers of R2C2 subreads used for polishing (--subreads).

To evaluate completeness and accuracy of the R2C2 subread, R2C2 consensus read and the final resulting Chopper-based full-length plasmid sequences we aligned these sequences to the respective plasmid reference sequences we downloaded from Addgene. Aligning circular sequences to each other can pose a challenge because the start of either experimentally determined or reference plasmid sequences will be at an arbitrary location in the plasmid. Aligning these to each other will therefore result in a primary (longer) and secondary (shorter) alignment. For our analyses, primary and secondary alignments of Chopper-based sequences to the Addgene reference sequence of each plasmid each were combined.

To assess completeness and overall error-rates first used the BLAT [12] aligner because it performed well when aligning sequences to the reference in cases where a secondary alignment would be very short. These alignments are shown in the circular plots in Fig 6A. These circular plots were generated based on the pslx files generated by BLAT using a custom script (CircularPlots.py).

For the analysis of exact accuracy of the different types of sequences, we used minimap2. Minimap2 alignment was run using the -ax map-hifi preset. We parsed the resulting sam file to retrieve the number of mismatches in each sequence alignment using a custom script (Sam2Identity.py). This script divides the number of mismatches by the overall length of the alignment to produce an overall error rate of each alignment which it then converts to identities [(1-error rate)*100]. These identities are shown for R2C2 subreads and R2C2 Consensus reads as swarmplots in Fig 3. These swarmplots are generated using a custom script (SwarmPlots.py).

For Chopper-based plasmid sequence, we determined that it was full-length and error-free if its length exactly matched its reference and its error rate was 0 for the purposes of Table 3.

To assess position dependent error-rates, we also ran the minimap2 using the --cs = long flag which causes minimap2 to produce a long cs string for each alignment which represents a pairwise alignment between sequence and reference. We parsed these long cs strings to evaluate the per- position error rates shown in Fig 6B using a custom script (PositionDependentError.py).

For Fig 6C, we aligned the sequences we received from Plasmidsaurus for the pSpCas9 plasmid to the pSpCas9 reference sequence using the EMBOSS WATER [13].

## Supporting information

S1 Fig Representative gel image of R2C2 DNA preparation. A 1% Agarose gel was run with R2C2 DNA after debranching of RCA product as part library preparation. The ladder on the left is a NEB 1kb+ ladder with the intense bands indicating 500, 1,000, and 3,000 bp and the highest band indicating 10,000 bp.This gel was generated as part of the development of the R2C2 method, not specifically for this study nor using plasmid DNA as input. However, the gel is representative of R2C2 DNA after debranching, regardless of sample input type.
(PDF)

S2 Data Supplemental scripts. (ZIP)
