# Supplementary material for: Sequencing complete plasmids on Oxford Nanopore Technologies sequencers using R2C2 and Chopper
Source: PLoS One. 2026 Apr 10;21(4):e0345168. doi: 10.1371/journal.pone.0345168 (PMC13068223; doi:10.1371/journal.pone.0345168)
Supplement: S1 Fig — A 1% Agarose gel was run with R2C2 DNA after debranching of RCA product as part library preparation. The ladder on the left is a NEB 1kb+ ladder with the intense bands indicating 500, 1,000, and 3,000 bp and the highest band indicating 10,000 bp.This gel was generated as part of the development of the R2C2 method, not specifically for this study nor using plasmid DNA as input. However, the gel is representative of R2C2 DNA after debranching, regardless of sample input type. (PDF) [file pone.0345168.s001.pdf]

Supplemental Data for

Sequencing complete plasmids on Oxford Nanopore Technology Sequencers using *R2C2* and *Chopper*

Kayla D. Schimke, Christopher Vollmers

Includes:

Fig. S1

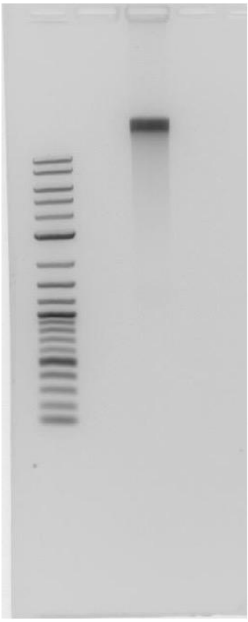

Fig S1: Representative Gel image of R2C2 DNA preparation. A 1% Agarose gel was run with R2C2 DNA after debranching of RCA product as part library preparation. The ladder on the left is a NEB 1kb+ ladder with the intense bands indicating 500, 1,000, and 3,000bp and the highest band indicating 10,000bp.

This gel was generated as part of the development of the R2C2 method, not specifically for this study nor using plasmid DNA as input. However, the gel is representative of R2C2 DNA after debranching, regardless of sample input type.
